# Diagnostic classification of solitary pulmonary nodules using dual time ^18^F-FDG PET/CT image texture features in granuloma-endemic regions

**DOI:** 10.1038/s41598-017-08764-7

**Published:** 2017-08-24

**Authors:** Song Chen, Stephanie Harmon, Timothy Perk, Xuena Li, Meijie Chen, Yaming Li, Robert Jeraj

**Affiliations:** 1 Department of Nuclear Medicine, The First Hospital of China Medical University, No. 155 North Nanjing Street, Heping District, Shenyang City, Liaoning Province 110001 P. R. China; 2 Department of Medical Physics, School of Medicine and Public Health, University of Wisconsin-Madison, University of Wisconsin Carbone Cancer Center, 7033 WIMR, 1111 Highland Ave, Madison, WI 53705 USA

## Abstract

Lung cancer, the most commonly diagnosed cancer worldwide, usually presents as solid pulmonary nodules (SPNs) on early diagnostic images. Classification of malignant disease at this early timepoint is critical for improving the success of surgical resection and increasing 5-year survival rates. ^18^F-fluorodeoxyglucose (^18^F-FDG) PET/CT has demonstrated value for SPNs diagnosis with high sensitivity to detect malignant SPNs, but lower specificity in diagnosing malignant SPNs in populations with endemic infectious lung disease. This study aimed to determine whether quantitative heterogeneity derived from various texture features on dual time FDG PET/CT images (DTPI) can differentiate between malignant and benign SPNs in patients from granuloma-endemic regions. Machine learning methods were employed to find optimal discrimination between malignant and benign nodules. Machine learning models trained by texture features on DTPI images achieved significant improvements over standard clinical metrics and visual interpretation for discriminating benign from malignant SPNs, especially by texture features on delayed FDG PET/CT images.

## Introduction

Solitary pulmonary nodules (SPNs) are common clinical findings, often incidental, that may represent malignant disease in the lung. SPNs are defined as a single, well defined pulmonary nodule with a diameter less than 3 cm and surrounded by normal lung tissue that is not associated with atelectasis or adenopathy^1^. Causes of SPN range from lung cancer and metastatic cancer to infections, scar formation, and other benign lesions. Previous studies showed that pulmonary nodules were detected in 69% of patients who underwent lung cancer screening with low-dose CT^2^, and 53% of SPNs were found to be malignant nodules^3^. Lung cancer usually presents as a SPN on diagnostic imaging at early stages of the disease^1^. Accurate classification of SPNs is important clinically, as diagnosis of malignant disease at this early timepoint is critical for improving the success of surgical resection and increasing 5-year survival rates.

^18^F- fluorodeoxyglucose (^18^F-FDG) PET has been demonstrated its utility for SPNs diagnosis with a high sensitivity to malignant SPNs detection; however, the application of FDG PET/CT is limited by its variable specificity estimates^4^. ^18^F-FDG, a PET tracer of glucose metabolism, has shown significant difference in uptake between malignant and benign lesions. Standard uptake value (SUV), the amount of tracer uptake inside the nodules, has been demonstrated good quantitative repeatability in lung nodules. Using SUV_max_ above 2.5 as a diagnostic threshold for malignant SPNs has been reported^5^, however, use of FDG PET/CT is less specific in diagnosing malignancy in populations with endemic infectious lung disease as compared with non-endemic regions. In order to improve the specificity of FDG PET/CT, some authors have proposed dual time point imaging (DTPI), using retention index (RI) to help differentiate benign and malignant SPNs. Unfortunately, the results of DTPI studies also showed varied sensitivity/specificity comparisons to single time point imaging, with both positive^6, 7^ and negative findings^8, 9^ being reported. There is an urgent need to improve the accuracy and specificity in diagnosing malignancy in populations from granuloma-endemic regions.

Beyond evaluating lesion SUV_max_, the spatial distribution of FDG also contains important information. Uptake of FDG is not homogeneously distributed within the lesions. Many factors, such as cellular proliferation, necrosis, blood flow and hypoxia, may contribute to intra-lesion heterogeneity^10^. Thus, measurements of this heterogeneity might help to distinguish benign from malignant pulmonary nodules.

Characterization of uptake heterogeneity is gaining popularity through radiomics-based analysis that extracts high throughput features based on intensity, shape, and texture of uptake within regions of interest. In CT imaging, the use of quantitative heterogeneity metrics for diagnostic purpose has been well studied, showing promising results in various cancer types^11, 12^. In FDG PET/CT, the use of texture features has improved discrimination between abnormal tissues from normal tissue for lesion delineation. Texture features derived from Neighboring Gray Tone Difference Matrix^13^, describing features such as coarseness, contrast and busyness on PET images have shown the ability to differentiate tumor from normal tissue in head and neck cancer^14^. However, there are only a few studies looking at the diagnostic value of quantitative heterogeneity features in FDG PET/CT imaging^15, 16^. Whether texture features can be able to discriminate malignant from benign lesions is still unknown. Furthermore, there are no studies evaluating the use of quantitative heterogeneity in DTPI PET/CT images for SPN differentiation.

This main goal of this study was to assesses the use of quantitative heterogeneity features extracted from DTPI images for differentiating malignant from benign SPN lesions in a population from granuloma-endemic regions. Machine learning models using texture features from DTPI PET/CT were tested and compared against commonly used clinical metrics and visual interpretation.

## Results

### Patient population

In total, 149 SPN patients underwent DTPI FDG PET/CT scans between 2004 and 2014 were reviewed. 64 patients were excluded from the study as the metabolic volume of the SPNs was smaller than 5 mL, making texture feature analysis unreliable. As a result, 85 patients (mean age: 61.58 ± 11.95, 56 male) were included in this study. Lesion diagnoses were confirmed by pathology (n = 74 lesions) or follow-up imaging (median: 14 months, range: 12–34 months) (n = 11 lesions). Sixty-three lesions were malignant nodules, with 61 confirmed by pathology results and 2 with evidence of metastasis in follow up period and subsequently clinically diagnosed as lung cancer. Twenty-two lesions were diagnosed to be benign lesions, 13 confirmed by pathology results, 4 diagnosed as stable nodules, 5 diagnosed as reduced nodules after treatment with antibiotics. Table 1 summarizes diagnoses of SPNs.

**Table 1:** Diagnosis of SPNs.

Type	Stage	Diagnosis	Number of cases
Benign			**22**
	Active inflammation		**17**
		Inflammatory pseudotumor	1
		Reduced nodule	5
		Tuberculosis and Granuloma	11
	Benign lung tumors		**1**
		Sclerosing hemangioma	1
	Old inflammation		**4**
		Stable nodules	4
Malignant			**63**
	Primary lung cancer		**60**
		Adenocarcinoma	37
		Large Cell Carcinoma	1
		Mucoepidermoidcarcinoma	2
		Unspecified NSCLC	6
		SCLC	7
		Squamous cell carcinoma	7
	Metastasis		**1**
		Thymic carcinoma	1
	Malignant nodules		**2**
		Unspecified malignant nodules	2

### Comparison of machine learning models and clinical metrics

**Figure 1:**
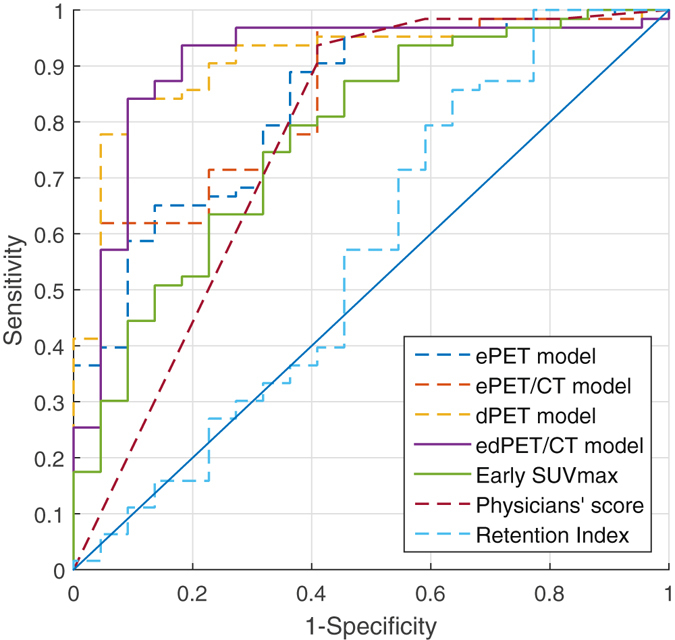
ROC curves of SVM models, early SUVmax, visual interpretation and retention index. Areas under curve showed the ability of machine learning models, early SUVmax, visual interpretation and retention index to distinguish malignant from benign SPNs. The dPET model and edPET/CT model had a significant improvement in discriminating power than early SUV_max_, visual interpretation and retention index.

The discriminating power of each model was compared by Area Under the Curve (AUC) of the Receiver Operating Characteristic curve (ROC) (Fig. 1). The ROC analysis showed that the AUC of standard clinical metrics, including early SUV_max_, delayed SUV_max_, RI, and visual interpretation were 0.77, 0.77, 0.56 and 0.76, respectively (Table 2). Of these clinical metrics, RI performed the worst with significantly lower AUC than both early SUV_max_ and visual interpretation (p = 0.01 and p = 0.02, respectively). Support Vector Machine (SVM) models of quantitative features showed AUCs from dPET (delayed PET) model and edPET/CT (early and delayed PET/CT) model of 0.90 and 0.91, respectively; which were significantly larger than AUC of either early SUV_max_ (P = 0.02, P = 0.01) or visual interpretation (P = 0.03, P = 0.04). Compared to either early SUV_max_ or visual interpretation, ePET (early PET) model and ePET/CT (early PET/CT) model showed moderate improvements, and eCT (early CT) model showed a little decline, but the differences were not significant (Table 2).

**Table 2:** AUC of ROC Analysis for each model.

Models	AUC	95%CI	P value^a^	P value^b^
eCT Model	0.72	0.58–0.84	0.47	0.59
ePET Model	0.83	0.74–0.93	0.14	0.27
ePET/CT model	0.83	0.74–0.93	0.14	0.24
dPET model^*^	0.90	0.83–0.97	0.02	0.03
edPET/CT model^*^	0.91	0.82–0.99	0.01	0.04
Early SUV_max_	0.77	0.66–0.89	—	0.88
Visual interpretation	0.77	0.65–0.88	0.88	—
RI	0.56	0.41–0.72	0.02	0.01

^*^P value smaller than 0.05.

^a^P value of Delong’s test, compare AUC of each model to that of early SUV_max_.

^b^P value of Delong’s test, compare AUC of each model to that of visual interpretation.

The performance of each model and indexes of the optimal thresholds were calculated and compared (Table 3). By using the optimal threshold, dPET and edPET/CT models achieved the best specificity, accuracy and positive predictive value. Two of the clinical indices, early SUV_max_ and RI, had a higher sensitivity than all SVM models, but the specificity was low.

**Table 3:** Diagnostic values for differentiation of malignant and benign SPN lesions with SVM models and indexes.

Models	True positive	True negative	Sensitivity	Specificity	Accuracy	PPV^*^	NPV^†^
ePET model	41	19	0.65	0.86	0.71	0.93	0.46
eCT model	39	16	0.62	0.72	0.65	0.87	0.40
ePET/CT model	45	17	0.71	0.77	0.73	0.90	0.49
**dPET model**	**53**	**20**	**0.84**	**0.91**	**0.86**	**0.96**	**0.67**
**edPET/CT model**	**53**	**20**	**0.84**	**0.91**	**0.86**	**0.96**	**0.67**
Physician’s Scores^**^ < 3.5	59	13	0.94	0.59	0.85	0.87	0.76
Early SUV_max_ > 2.5	62	4	0.98	0.18	0.78	0.78	0.80
RI > 10%	59	5	0.94	0.23	0.76	0.78	0.63

^*^Positive predictive value †, Negative prediction value, **Scores of visual interpretation.

Table 4 summarizes the frequency of features selected by models more than 2 times. Delayed Busyness had the highest frequency of selection, being chosen in all 5/5 cross validations of the combined edPET/CT model and 3/5 dPET models. The most commonly selected feature-types are “Busyness”, “Coarseness” and “Cluster Prominence” on either delayed or early PET.

**Table 4:** Frequency of most commonly selected features for each model.

	CT	ePET	ePET/CT	dPET	edPET/CT*	Total
Busyness	—	2	4	3	0/5	14
Cluster Prominence	—	3	1	4	1/2	11
Coarseness	—	2	2	5	1/0	10
Sum Variance	—	1	2	4	0/0	7
Coefficient Of Variation	—	3	3	—	0/0	6
Standard Deviation	—	2	3	—	0/0	5
CT Skewness	0	—	4	—	1	5
CT Entropy	3	—	1	—	1	5
CT Busyness	3	—	1	—	0	4
CT Long Run High Gray Level Emphasis	2	0	1	—	0	3
Maximal Correlation Coefficient	—	0	0	1	0/2	3
Run Percentage	—	0	0	1	0/2	3
CT Diagonal Moment	1	—	1	—	1	3

Note: maximum selected in each model is equal to the number of cross-validations performed (5).

*For edPET/CT model, frequency of PET features is represented as “early PET features”/“delayed PET feature”, representing number of times “early PET features” was selected and number of times “delayed PET feature” was selected.

## Discussion

In this study, we were able to demonstrate significant improvement in classifying malignant from benign SPNs using machine learning techniques. The machine learning algorithms were trained on quantitative heterogeneity features from DTPI PET and CT images and results were shown to be superior to commonly used clinical metrics and visual interpretation. Using an SVM model created with DTPI FDG PET/CT features, we were able to achieve higher diagnostic accuracy, marked by higher specificity and sensitivity, than commonly used clinical metrics and visual interpretation. This is the first report of using texture features for diagnosis of solitary pulmonary nodules, and the first study to evaluate texture features in delayed PET images. The improvement in diagnostic performance shown in this study could potentially benefit patients by preventing unnecessary invasive tests following false-positive findings or providing earlier detection and intervention in patients with malignant disease.

We compared the diagnostic accuracy of SVM models with that of two experienced physicians. The diagnostic ability of SVM created from multiple early FDG PET/CT texture features was comparable to two experienced physicians. With the addition of delayed PET features, the SVM models showed greater diagnostic accuracy compared to physician assessment and common clinical metrics. Therefore, SVM analysis from DTPI FDG PET/CT might play an integral role as a semi-automated tool to supplement diagnostic classifications for physician readers.

Previous studies have reported success using features from high quality diagnostic CT in classification of lung cancer^12^. Results from our study suggest CT texture features from free breath CT images of FDG PET/CT may not provide more information than PET features along for classification purposes. Models using only CT features can be used in SPNs classification, but the differentiation ability is weak, and no improvements were seen compared to using only early SUV_max_. Additionally, the AUC and accuracy for the ePET model and the ePET/CT model are similar, suggesting that the addition of early CT features to early PET features did not improve the diagnosis ability significantly.

FDG PET/CT has been demonstrated as a useful, yet incomplete, tool for SPN diagnosis. One of the main limitations is that many benign lesions demonstate high FDG uptake which lead to false-positive results. Active infectious and non-infectious inflammatory etiologies can lead a high uptake in FDG PET. There are numerous reports in the literature of false-positive findings on FDG PET imaging due to granulomas and tuberculosis^17^. Use of FDG PET/CT was less specific in diagnosing malignant lesions in populations with endemic infectious lung disease compared with non-endemic regions^4^. The average adjusted specificity in regions with endemic infectious lung disease is 16% lower, compared with nonendemic regions^4^. Improving the accuracy and specificity of FDG-PET/CT in diagnosing malignancy especially in populations from granuloma-endemic regions, which would be expected to have a higher rate of false-positive FDG PET/CTs due to uptake in benign inflammatory granulomas, is a difficult problem. In this study, both visual interpretation and early SUV_max_ had a low specificity and accuracy in diagnosis malignant SPNs, but SVMs using texture features extracted from both timepoints in DTPI FDG PET/CT images achieved higher specificity and accuracy over clinical metrics and visual interpretation. This support the hypothesis that intratumoral heterogeneity of FDG uptake was useful for discriminating benign from malignant nodule for the patients from granuloma-endemic regions.

## Materials and Methods

This retrospective study was approved by the Medical Science Ethic Committee of the 1^st^ hospital of China Medical University, and formal consent was not required. All procedures performed in studies involving human participants were in accordance with the ethical standards of the institutional. This study was retrospective, and its results did not influence further therapeutic decision-making.

### Patients

DTPI ^18^F-FDG PET/CT scans were performed between 2004 and 2014 at a single center in a granuloma-endemic region. The scans were retrospectively reviewed and those scans with a Single Pulmonary Nodule (SPN) were analyzed. Diagnosis of malignant or benign disease was primarily established by pathology review following biopsy or surgical resection of the nodule within one month after the FDG PET/CT acquisition. When biopsy or surgery was not performed, patients were followed for at least 1 year after the FDG PET/CT scan, using chest radiography or CT. If the SPN was stable during this period or reduced with antibiotics treatment, they were considered benign. Patient without pathological confirmation or those receiving surveillance follow-up imaging less than 1 year were removed from this study. In total, FDG PET/CT data from 149 patients was reviewed under these criteria.

### Image acquisition and reconstruction

Patients fasted for at least 6 h, and blood glucose levels were measured before being injected with 5.55MBq/kg (0.15 mCi/kg) ^18^F-FDG. Early and Delayed FDG PET/CT acquisition started 60 min and 180 min post injection using a GE discovery LS 4 PET/CT scanner. Emission data were acquired for 3 min per bed position in 2D mode. The PET images were reconstructed using an iterative algorithm (ordered-subsets expectation maximization: 2 iterations, 28 subsets) with an 8-mm Gaussian filter, a 128 × 128 matrix and 4.25 mm/slice. Transmission scanning proceeded under the following parameters.

### Image Analysis

Nodules on both early FDG PET (ePET) and delayed FDG PET (dPET) images were identified and segmented by two experienced physicians (with more than 5 years experiences working in nuclear medicine). Discordant segmentations were resolved by discussion and mutual consensus. Lesions with volume of interest (VOI) smaller than 5 mL on the early FDG PET/CT images were removed from this study, following recommendations from previous studies showing FDG PET texture features uncertainty in small volumes^18, 19^.

For each lesion, two quantitative clinical metrics, SUV_max_ and Retention Index(RI), were calculated.

RI was calculated according to equation (1).

1 $${\rm{RI}}=100 \% \times \frac{({\rm{delayed}}\,{{\rm{SUV}}}_{{\rm{\max }}}-{\rm{early}}\,{{\rm{SUV}}}_{{\rm{\max }}})}{{\rm{early}}\,{{\rm{SUV}}}_{{\rm{\max }}}}$$

Additionally, for each lesion in this study, a visual interpretation score was made based on DTPI FDG PET/CT images (both early and delayed FDG PET/CT images). All images were interpreted by two physicians. Image interpretation was performed without knowledge of patient diagnosis to avoid potential interpretation bias. A 5-point scale was used when readers interpreted the images, which represent the likelihood of the lesion being benign or malignant, from: 1, definitely benign; 2, probably benign; 3, equivocal; 4, probably malignant; and 5, definitely malignant. If the interpretation scores were discordant between two readers, they were subsequently discussed to arrive to a consensus.

The following interpretive criteria^20^ were utilized:

#### CT image interpretation

Each nodule was characterized in terms of its attenuation, shape, and margin characteristics using previously well-described criteria developed by the American College of Radiology Imaging Network (ACRIN) for its lung cancer screening trials^21, 22^.

#### FDG PET image interpretation

FDG PET images interpretation was based on the degree and the distribution of the uptake in the lesion on the early FDG PET images as well as the contrast changes apparent on the delayed FDG PET image. If lesion uptake was higher than blood pool in the early FDG PET and appeared distinctly focal and its contrast increased in delayed FDG PET, the lesion was categorized as more likely to be malignant. In contrast, if the lesion was poorly defined with a low uptake in the early FDG PET and lost contrast in delayed FDG PET, it was interpreted as more likely to be benign. On the basis of integration of FDG PET characteristics (uptake degrees, distribution of uptakes, spatial volume effect, and contrast changes) and CT characteristics (attenuation, shape, and margin characteristics) the readers then made a 5-point scale score.

### Quantitative Texture-based Analysis

Texture features were extracted following voxel-based methodology described previously^23, 24^. Uptake values contained within the Volume of Interest (VOI) were resampled prior to texture feature extraction using a 256-bin discretization. For each voxel inside the VOI, a patch was extracted, defined as a portion of the image with 5 × 5 × 5 (axial, coronal, sagittal) voxels in size, centered on that voxel. Texture features were computed on these patches in 4 angular directions on axial, coronal and sagittal slices and then the mean value was calculated. For each lesion, 59 features were extracted respectively on early FDG PET images (ePET), delayed FDG PET images (dPET) and CT (CT) images with an in-house code developed in MATLAB, using methodology as described by Galavis *et al*.^24^, adapted to include all nearest neighbors of each voxel. This methodology of 3D extraction was motivated by previous works showing increasing the number of neighboring planes did not increase observed performance of extracted spatial information^25, 26^. Those features included: 10 histogram based first order features, eight first order features, 22 features based on the co-occurrence matrix, 11 features based on the gray level run length matrix, 5 features based on the neighboring gray level and 3 features based on the neighborhood gray tone difference matrix (Table 5).

**Table 5:** Texture features.

Image Feature Basis	Features
Histogram	Max, Total Lesion Glycolysis, Mean, Min, Volume, Skewness, Kurtosis, Energy, Entropy, Standard Deviation
First order features	Mean, Median, Coefficient of Variation, Skewness, Kurtosis, Energy, Entropy, Variance
Co-occurrence matrix	Angular Moment, Contrast-GLCM, Correlation, Sum of Squares Variance, Inverse Difference Moment, Sum Average, Sum Variance, Sum Entropy, Entropy-GLCM, Difference Variance, Difference Entropy, Information Measure of Correlation 1, Information Measure of Correlation 2, Maximal Correlation Coefficient, Maximum Probability, Diagonal Moment, Dissimilarity, Difference Energy, Inertia, Inverse Difference Moment, Sum Energy, Cluster Shade, Cluster Prominence
Gray level run length	Small Run Emphasis, Long Run Emphasis, Gray-Level Nonuniformity, Run Length Nonuniformity, Run Percentage, Low Gray-Level Emphasis, High Gray-Level Emphasis, Short Run Low Gray-Level Emphasis, Short Run High Gray-Level Emphasis, Long Run Low Gray-Level Emphasis, Long Run High Gray-Level Emphasis
Neighboring gray level	Small Number Emphasis, Large Number Emphasis, Number Nonuniformity, Second Moment, Entropy-NGL
Neighborhood grey tone difference matrix	Coarseness, Contrast-NGL, Busyness

In total, 177 features were calculated for each lesion: 59 features from ePET images, 5, 59 features from dPET images and 59 features from CT images.

### Features selection and testing machine learning models

Five-fold cross validation was used in this study to divide the original data into training data and validation data. By using 5-fold cross-validation^27^, the original data is randomly partitioned into 5 equally sized subsamples. Of the 5 subsamples, a single subsample is retained as the validation data for testing the model, and the remaining 4 subsamples are used as training data. The cross-validation process is then repeated 5 times, with each of the 5 subsamples used exactly once as the validation data. The 5 results from the folds can then be averaged to produce a single estimation.

In order to reduce the size of the dataset, sequential forward floating selection (SFFS) was used to select only a few critical features to training the Support Vector Machine (SVM) models^28^. The SFFS was performed in the training dataset and the maximal number of selected features was set to five. Five SVM models were built with selected features from different feature sets: (1) ePET model: early PET features, (2) eCT model: early CT features, (3) dPET model: delayed PET features, (4) ePET/CT model: early PET/CT features, (5) edPET/CT model: early PET/CT features and delayed PET features. The SVM models were built by several built-in functions in Matlab (ver.2015b) using the default parameters.

The performance of each machine learning model to perform classification of unseen SPNs lesion into the benign or malignant was tested using receiver operating characteristics (ROC) analysis.

### Comparison the performance of models with clinical metrics and visual interpretation

The performance of each model (SVM models, clinical metrics and visual interpretation) was evaluated using the areas under ROC curves (AUC), diagnostic accuracy, sensitivity, specificity, positive prediction value, and negative prediction value. AUCs were compared using Delong’s test^29^. Values plotted nearest the upper left corner of the ROC plot were considered to be the optimal threshold for diagnosis. The diagnostic accuracy, sensitivity, specificity were calculated using the optimal threshold and commonly accepted clinical metrics (early SUV_max_ > 2.5 and RI > 10% for malignant lesions).

## Conclusions

The intratumoral heterogeneity of FDG uptake was useful for discriminating benign from malignant nodules in larger SPNs especially on delayed PET images for the patients from granuloma-endemic regions. Texture features on FDG DTPI provided different types of information that should be used to supplement SUV_max_ for making a diagnosis. SVMs and texture features extracted from DTPI FDG PET/CT images showed a significant improvement in discriminating benign from malignant nodules over commonly used clinical metrics and visual interpretation.
